# Trends in the Rates of Pediatric Pyeloplasty for Ureteropelvic Junction Obstruction over 19 Years: A PHIS Database Study

**DOI:** 10.1155/2014/142625

**Published:** 2014-05-13

**Authors:** Ardavan Akhavan, Paul A. Merguerian, Cindy Larison, Adam B. Goldin, Margarett Shnorhavorian

**Affiliations:** ^1^Division of Pediatric Urology, Seattle Children's Hospital, 4800 Sand Point Way, Seattle, WA 98105, USA; ^2^Seattle Children's Research Institute, 1900 Ninth Ave, Seattle, WA 98101, USA; ^3^Department of Surgery, Seattle Children's Hospital, 4800 Sand Point Way, Seattle, WA 98105, USA

## Abstract

*Background*. Over the past 20 years, the management of ureteropelvic junction obstruction (UPJ) has shifted. While many urologists note a decrease in the number of pyeloplasties performed over time, the nature of the change in practice has yet to be defined. In the current study, we utilize a national, multi-institutional database of children's hospitals to evaluate trends in patients undergoing pyeloplasty as well as the rate of surgical reconstruction over the past 20 years. *Material/Methods*. We queried the Pediatric Health Information System (PHIS) database for all children undergoing primary pyeloplasty between 1992 and 2011. Clinical variables, including age at time of surgery, gender, length of stay (LOS), and geographic region, were determined. Age-adjusted rate of repair was also calculated per 100,000 PHIS inpatients. *Results*. 6,013 patients were included in the study, of which 71.6% were male and 64.2% were under the age of 24 months at time of surgery. Over the study period, the median age at time of surgery increased from 2–4 months to 12–14 months (*P* < 0.01). LOS decreased from a median of 5 days to 2 days (*P* < 0.001). The rate of surgery increased by 10.6 pyeloplasties per 100,000 PHIS inpatients from 1992 to 2011 (*P* < 0.01). The highest rate of pyeloplasty was in the northeast. The increase in pyeloplasties performed from 1992 to 1999 was specific to children aged greater than 24 months, while rates stayed the same in infants younger than 2 years during the same time period. In contrast, from 1999 to 2011, the rate of pyeloplasty decreased in patients less than 2 years of age, while the rate remained constant in patients over age 2. *Conclusion*. The rate of pyeloplasty increased in PHIS hospitals from 1992 to 2011. Trends are due to an increase in surgery in infants younger than 2 years from 1992 to 1999, followed by a progressive surgical rate decline, characterized by a shift towards patients older than 2 years of age.

## 1. Introduction

The management of congenital ureteropelvic junction (UPJ) obstruction has evolved over the past 20 years. Following the advent of prenatal ultrasound screening, initial excitement over early repair of UPJ obstruction [1, 2] has been tempered by numerous studies demonstrating a mostly benign natural history [3–14]. While pediatric urologists had initially recommended early treatment for all children with evidence of obstruction [1], surgery is now reserved for those with decreasing renal function, recurrent infection, flank pain, or worsening hydronephrosis. Using this criterion, contemporary studies demonstrate that only 22% of children with unilateral hydronephrosis [3] and 35% with severe bilateral hydronephrosis eventually undergo reconstruction [14].

The effect of this conservative shift in philosophy towards the management of UPJ obstruction is unclear. While many surgeons note anecdotal decreases in the volume of surgeries performed for pediatric UPJ obstruction, overall longitudinal trends in practice patterns have not been formally evaluated since 2005 [15]. In the current study, we examined trends in the rates of both open and minimally invasive pyeloplasties performed in children and adolescents with UPJ obstruction over the past 19 years, by examining a nationwide, multi-institutional pediatric hospital database.

## 2. Data and Methods

Data for this study were obtained from the Pediatric Health Information System (PHIS), an administrative database that contains inpatient, emergency department, ambulatory surgery, and observation data from 43 not-for-profit tertiary care pediatric hospitals in the United States. These hospitals are affiliated with the Children's Hospital Association (Overland Park, KS). Data quality and reliability are assured through a joint effort between the Children's Hospital Association and participating hospitals. The data warehouse function for the PHIS database is managed by Truven Health Analytics (Ann Arbor, MI). For the purposes of external benchmarking, participating hospitals provide discharge/encounter data including demographics, diagnoses, and procedures. Forty-two of these hospitals also submit resource utilization data (e.g., pharmaceuticals, imaging, and laboratory) into PHIS. Data are deidentified at the time of data submission, and data are subjected to a number of reliability and validity checks before being included in the database. For this study, data from 43 hospitals was included.

Approval was obtained from the Seattle Children's Institutional Review Board (number 13630). Inclusion criteria consisted of all inpatients aged 0–19 years and treated between 1992 and June 30, 2011, who had a principal ICD-9 procedure code for correction of ureteropelvic junction (55.87) and principal ICD-9 diagnosis code for congenital obstruction of ureteropelvic junction (753.21). Laparoscopic and robotic pyeloplasties performed between 2004 and 2011 were identified using ICD-9 procedure codes 17.42 and 5421, respectively. Patients who had repeated procedures and those who underwent endoscopic UPJ repair were excluded. Results were blinded to hospital identity, which is consistent with PHIS policies.

The main outcome variables were the timing (age) of pyeloplasty and overall and age-adjusted incidence rates of pyeloplasty. We also looked at gender, length of stay (LOS), and geographic region of treatment.

## 3. Analysis

Analysis was performed using STATA SE v12 software (StataCorp., College Station, TX). The significance level for all tests was set at *P* < 0.05. Connected scatter plots were used to examine the data. Because of the skewed distribution of age in months, Cuzick's nonparametric test of trend was used to assess a trend in timing over years. In addition, we used analysis of variance (ANOVA) to test the trend on the natural logarithm of age in months.

The number of pyeloplasties was calculated for each year in the study period for patients of all ages. To adjust for the increasing number of patients in the database, the rate of repair was derived by dividing the number of pyeloplasties each year by the total number of patients encountered by PHIS hospitals for the year of repair and expressed as the number of pyeloplasties per 100,000 patients in PHIS. The yearly rate summaries were analyzed for trend by both Cuzick's and ANOVA tests for trend.

The analysis of the relationship of year to rate of repair controlling for timing was assessed using linear regression. While we used data for each year separately in the analysis, in some of the graphic depiction of this data we combined years into groups of 4 for ease of viewing. Patients were stratified by age into two groups: younger than 24 months and over 24 months.

## 4. Results

**Figure 1:**
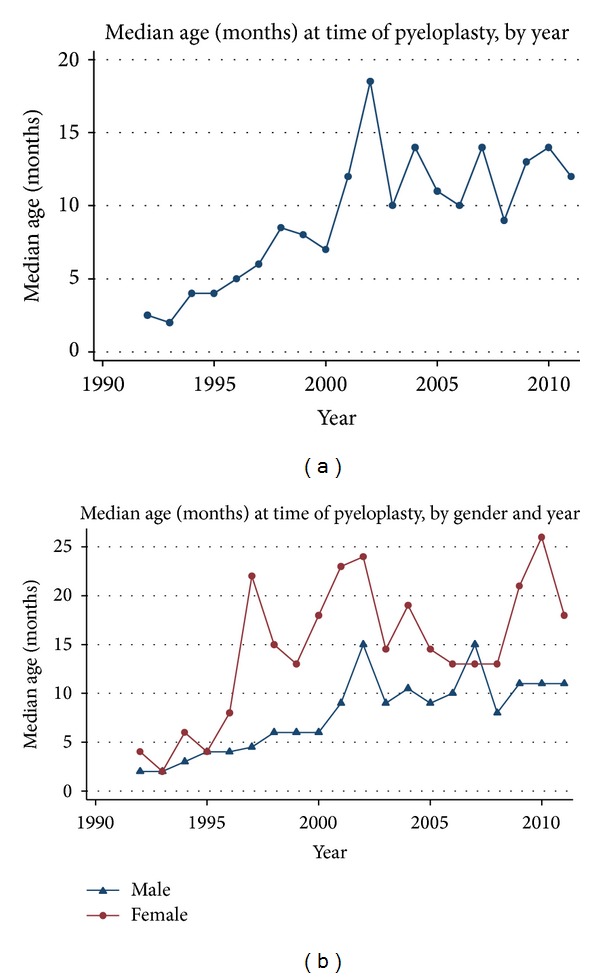


Our analysis screened 6,157 pyeloplasties, which was narrowed down to 6,013 patients after exclusion of 144 (2.3%) redo procedures. Infants under 24 months of age made up the majority (64.2%) of the undergoing surgeries. Analysis of timing of surgery shows an increase in age over the entire time period, with median age between 2 and 4 months in 1992–1995, increasing to a median age of 12–14 months in 2009–2011 (*P* < 0.01) (Figure 1(a)).

Overall, 71.6% of patients were male. The proportion of male patients ranged from 67.4% to 78.6% over the 19.5-year period; the trend was not significant.  Figure 1(b) demonstrates the change in timing of surgery over the evaluation period by gender. The median age at surgery for males and females was consistent before 1996 (boys = 4 months, girls = 8 months). After 1996, the median age at surgery increased for both boys and girls. Overall, the trend of increase was significant for both genders (boys *P* < 0.01, girls *P* < 0.05).

**Figure 2:**
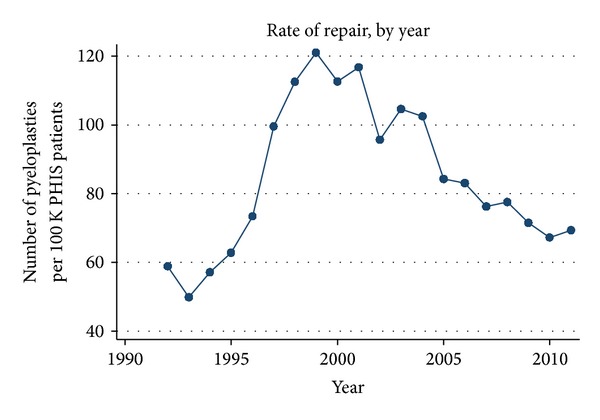


**Figure 3:**
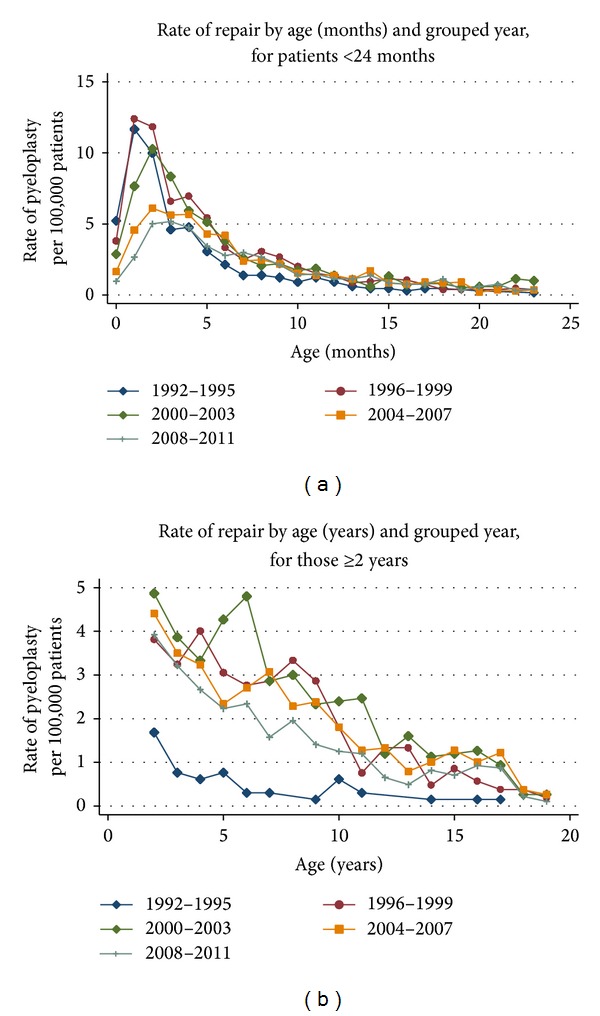


Figure 2 shows an upward trend in the rate of repair from 58.8 pyeloplasties per 100,000 PHIS inpatients in 1992 to as high as 121 in 1999 and then a steadily decreasing trend to a rate of 69.4 in the first half of 2011. When controlling for age, linear regression analysis demonstrates this change to be significant (*P* < 0.001). The highest rates of repair were in patients less than 6 months of age. Figures 3(a) and 3(b) show rates of repair by age for patients under 2 years old and over 2 years old, respectively. There is an increase in the rate of pyeloplasty performed after 1995, particularly in patients of 3 months of age. Surgery shifts towards older children, though, as a decline in the rate of reconstruction in infants younger than 2 years of age is associated with an increase in the rate of surgery in children over the age of 2.

**Figure 4:**
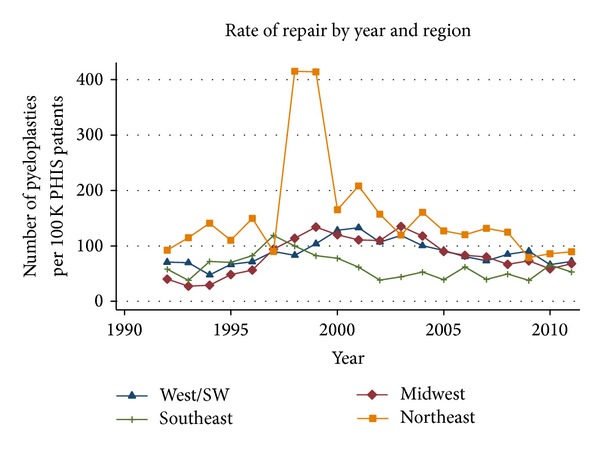


Figure 4 illustrates the rate of repair by year, categorized by region. Over the entire study period, the highest rate of pyeloplasty was in the northeast. Interestingly, the rates in the northeast peaked in 1998 and 1999, when the number of repairs dramatically spiked to 415.4 and 413.8 per 100,000 PHIS patients, respectively. This pattern was not seen in any other regional group; thus, the 1999 peak in pyeloplasties demonstrated in Figure 2 appears to be solely due to trends in the northeast.

**Figure 5:**
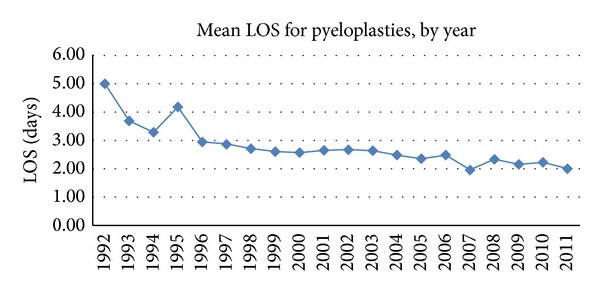


Figure 5 illustrates mean length of stay (LOS) following pyeloplasty from 1992 to 2011. Over the study period, median LOS decreased significantly from 5 days to 2 days (*P* < 0.001).

Table 1 illustrates the proportion of laparoscopic and robotic pyeloplasties performed between 2004 and 2011. During this 7-year period, a total of 61 (2.1%) robotic and 120 (4.1%) laparoscopic pyeloplasties were performed in infants and children at PHIS hospitals. While the rate of robotic pyeloplasty has been relatively constant, the proportion of laparoscopic pyeloplasties has steadily increased since 2008.

## 5. Discussion

Our study demonstrated an overall increase in the rate of infant pyeloplasties performed from 1992 to 1999, followed by a steady decline since. Interestingly, despite the conception that the rate of pyeloplasties has decreased, the net rate of surgical reconstruction of congenital UPJ obstruction has actually increased from 58.8 pyeloplasties per 100,000 PHIS inpatients in 1992 to 69.4 pyeloplasties per 100,000 PHIS inpatients in 2011. Our geographic analysis demonstrates that trends in the northeast were primarily responsible for the spike in the rate of pyeloplasties in 1999 (Figure 4). The reasons for this geographic discrepancy are unclear.

Our analysis also demonstrates an increase in infant pyeloplasty from 1992 to 1999, followed by a progressive decrease in the rate of surgery in these children in subsequent years. The latter decrease in infant surgical rates was associated with an increase in surgical rates in older children. These results are comparable to those of Nelson et al. who queried the Nationwide Inpatient Sample (NIS) database and discovered a proportional decrease in the rate of pyeloplasty performed in children under 6 months of age from 1988 to 2000 [15]. The most likely reason for this trend towards older pyeloplasty is progressive acceptance of studies supporting observation for most children with hydronephrosis throughout the 1990s [3–14], thereby tempering the traditional dogma that all hydronephrosis represented significant obstruction and must be treated immediately following diagnosis in order to prevent renal damage [1]. Many of the same children are still getting surgery; however, they are undergoing surgery at an older age. The shift towards surgery in older children is also likely a reflection of the widespread adoption of minimally invasive pyeloplasty during the study time period.

An increasing proportion of cases are being performed using minimally invasive methods. The ability to evaluate robotic cases prior to 2004 is limited by the lack of specific CPT coding available until that time. However, we noted that 4.1% and 2.1% of pyeloplasties performed between 2004 and 2011 were performed laparoscopically and robotically, respectively. These rates are comparable to those of Vemulakonda et al., who reported the results of a PHIS database inquiry and found a 7.5% rate of minimally invasive pyeloplasty [16]. Similarly, Monn et al. reported their results of an NIS database search and reported an 8.9% rate of minimally invasive pyeloplasty performed between 2005 and 2010 [17]. With the widespread adoption of robotic modalities, the proportion of minimally invasive pyeloplasty is sure to increase, particularly in older children; however, this increase does not appear to translate into an overall increase in pyeloplasty rates, as our study confirms a continued decrease in the rate of pyeloplasties performed over the past 12 years.

We also identified a significant decrease in the LOS following pyeloplasty, with the median LOS dropping from 5 days in 1992 to 2 days in 2011. Figure 5 demonstrates a steady decrease in LOS following pyeloplasty beginning in 1993. This trend is corroborated by the study by Nelson et al., who confirmed a decreasing LOS over time, and also demonstrated an association with insurance type, comorbidities, race, socioeconomic status, and academic status of the treating hospital [15]. While the adoption of laparoscopy and robotics is often also credited for this shortening of hospitalization, it should be noted that Vemulakonda et al. found no significant difference in mean LOS between patients undergoing minimally invasive pyeloplasty and those having open surgery [16]. The shortening hospitalization may be due to other generalized factors leading to well-recognized decreases in LOS among multiple subspecialties over the last two decades, including use of care pathways, local legislation, and changing surgical practices [15, 18–21].

The current study has several limitations that warrant recognition. PHIS is an administrative database containing information from 43 free-standing children's hospitals across the United States. The practices utilized by these tertiary care centers are not necessarily representative of the rest of the hospitals in the nation. Additionally, given the retrospective nature of the study, we identified patients and procedures using ICD-9 codes; theoretically, improperly coded patients and those treated as outpatients may be missed by this retrospective evaluation. However, prior studies have demonstrated a 96–98% coding accuracy for PHIS data [22]. Additionally, the study failed to differentiate patients treated with minimally invasive surgery prior to 2004. This was due to the lack of ICD-9 procedure codes related to robotic and minimally invasive surgery prior to that time. Vemulakonda et al. were able to capture this data by looking up charges specific to minimally invasive surgery in order to identify fewer than 100 minimally invasive pyeloplasties performed in PHIS hospitals prior to 2004 [16]. Additionally, given the shortcomings of large database studies, we are limited in our conclusions, as we do not know presenting symptoms and cannot determine reasons for surgical decisions.

## Figures and Tables

**Table 1:** 

Year	Number of pyeloplasties	Number of PHIS patients	Rate per 100,000	Number of lap. pyeloplasties	Number of robotic pyeloplasties
2004	446	435154	102.49	0 (0%)	12 (2.7%)
2005	395	468612	84.29	0 (0%)	1 (0.3%)
2006	395	475487	83.07	0 (0%)	3 (0.8%)
2007	385	504858	76.26	0 (0%)	13 (3.4%)
2008	398	512779	77.62	8 (2%)	10 (2.5%)
2009	383	535835	71.48	32 (8.4%)	5 (1.3%)
2010	350	520587	67.23	48 (13.7%)	11 (3.1%)
2011*	185	266726	69.36	32 (17.3%)	6 (3.2%)

2004–2011	2937	3720038	78.95	12 (4.1%)	61 (2.1%)

*Data from 2011 only through June 30, 2011.
